# Supplementary material for: Time-resolved burst variance analysis
Source: Biophys Rep (N Y). 2023 Jul 7;3(3):100116. doi: 10.1016/j.bpr.2023.100116 (PMC10406964; doi:10.1016/j.bpr.2023.100116)
Supplement: Document S1. Figure S1 [file mmc1.pdf]

Biophysical Reports, Volume *3*

Supplemental information

Time-resolved burst variance analysis

Ivan Terterov, Daniel Nettels, Dmitrii E. Makarov, and Hagen Hofmann

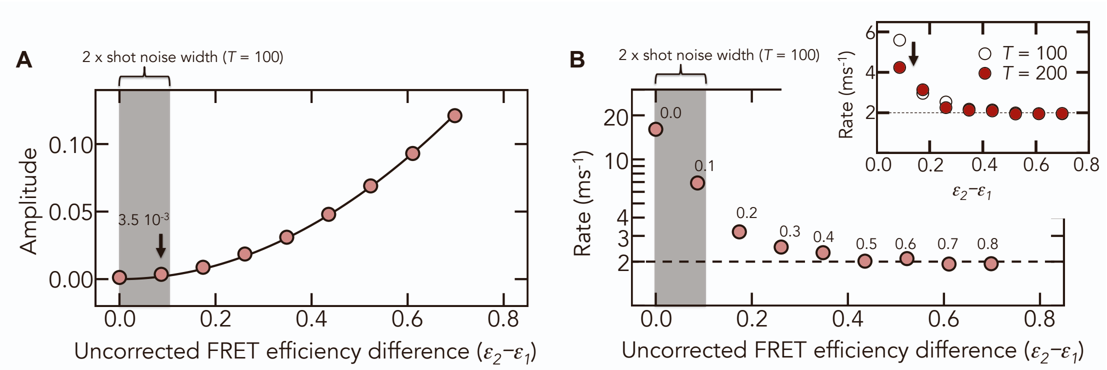

Figure S1. Simulations of 2-states in exchange with different FRET efficiency separation. (A) Amplitudes of the FRET autocorrelation function as function of the uncorrected FRET efficiency separation between the two states. The exchange rates were $k_{12} = k_{21} = 1ms^{-1}$ and the positions of the states were at the corrected FRET efficiencies $E_1 = 1/2 - \Delta E$ and $E_2 = 1/2 + \Delta E$. The solid line is the expected increase based on eq. 7 in the main text. The gray area indicates the regime of strong overlap between the two FRET states (see eq. VI.1). **(B)** Kinetic rates as function of the uncorrected FRET efficiency separation between the states obtained from the simulations shown in A. The dashed line indicates the ground truth ($k_{12} + k_{21} = 2ms^{-1}$). Numbers above the symbols indicate the corrected FRET efficiency separation ($2\Delta E$). The gray area indicates the regime of strong overlap between the two FRET states (see eq. VI.1). Inset: Dependence of the kinetic rate on the choice of the burst identification threshold T (indicated).
