# Supplementary material for: Time-resolved burst variance analysis
Source: Biophys Rep (N Y). 2023 Jul 7;3(3):100116. doi: 10.1016/j.bpr.2023.100116 (PMC10406964; doi:10.1016/j.bpr.2023.100116)
Supplement: Document S2. Article plus supporting material [file mmc2.pdf]

Biophysical Reports

ARTICLE

Time-resolved burst variance analysis

Ivan Terterov,[1] Daniel Nettels,[2] Dmitrii E. Makarov,[3] and Hagen Hofmann[1,*]

[1]Department of Chemical and Structural Biology, Weizmann Institute of Science, Rehovot, Israel; [2]Department of Biochemistry and Department of Physics, University of Zurich, Zurich, Switzerland; and [3]Department of Chemistry and Oden Institute for Computational Engineering and Sciences, University of Texas at Austin, Austin, Texas

ABSTRACT Quantifying biomolecular dynamics has become a major task of single-molecule fluorescence spectroscopy methods. In single-molecule Förster resonance energy transfer (smFRET), kinetic information is extracted from the stream of photons emitted by attached donor and acceptor fluorophores. Here, we describe a time-resolved version of burst variance analysis that can quantify kinetic rates at microsecond to millisecond timescales in smFRET experiments of diffusing molecules. Bursts are partitioned into segments with a fixed number of photons. The FRET variance is computed from these segments and compared with the variance expected from shot noise. By systematically varying the segment size, dynamics at different timescales can be captured. We provide a theoretical framework to extract kinetic rates from the decay of the FRET variance with increasing segment size. Compared to other methods such as filtered fluorescence correlation spectroscopy, recurrence analysis of single particles, and two-dimensional lifetime correlation spectroscopy, fewer photons are needed to obtain reliable timescale estimates, which reduces the required measurement time.

WHY IT MATTERS Single-molecule fluorescence spectroscopy, particularly in combination with Förster resonance energy transfer, has been extremely successful in quantifying the dynamics of biomolecules. A toolbox of different methods is available to date that extracts dynamic information from the stream of photons emitted from donor and acceptor dyes. Yet, some of these methods require long integration times. In others, the presence or absence of dynamics is difficult to judge by eye and only fits with kinetic models provide this information. We therefore extended the popular method of burst variance analysis (BVA) to overcome some of these limitations. The new method termed time-resolved BVA quantifies dynamics from 5 μs to 5 ms at high accuracy with as little as 5000 bursts. Static and dynamic heterogeneity can be distinguished from each other, and even dynamics slower than the diffusion time can be quantified. Time-resolved BVA is a natural extension of classical BVA and therefore easy to implement by researchers in the field of single-molecule Förster resonance energy transfer.

INTRODUCTION

The flexibility of proteins is key for their function. Resolving structural heterogeneity and quantifying the timescales at which proteins interconvert between different structural states has been a major goal in single-molecule fluorescence spectroscopy (1–4). Single-molecule Förster resonance energy transfer (SmFRET) has particularly been used in the past 2 decades to study conformational changes in biomolecules (5,6). Most smFRET experiments use freely diffusing molecules. These experiments are easy to realize and avoid tethering of molecules to surfaces. Naturally, a range of methods has been developed to extract dynamic information during the time molecules reside in the excitation volume of a confocal microscope (~1 ms). These methods range from dynamic photon distribution analysis (7), over maximum likelihood approaches (8–12) and equivalent Hidden-Markov model fitting such as H^2MM (13,14) and multiparameter H^2MM (15), fitting of FRET-histograms with different time binning (16), lifetime-filtered fluorescence correlation spectroscopy (fFCS) (17,18), two-dimensional lifetime correlation spectroscopy (19–21), recurrence analysis of single particles (RASP) (22,23), and lately a particularly promising approach using Bayesian nonparametrics (24–26). Each method has its merits and pitfalls. For instance, H^2MM and maximum likelihood directly use the photon arrival times to optimize the parameters of a

Submitted May 3, 2023, and accepted for publication July 3, 2023.

*Correspondence: hagen.hofmann@weizmann.ac.il

Editor: Jorg Enderlein.

https://doi.org/10.1016/j.bpr.2023.100116

© 2023 The Authors.

This is an open access article under the CC BY license (http://creativecommons.org/licenses/by/4.0/).

kinetic model and capture dynamics over a broad range of timescales. Dynamic photon distribution analysis computes FRET efficiency histograms by integrating the probability density that a molecule spends a certain time in each state of a kinetic model. The fit quality in these methods is often judged by generating FRET distributions from the model fit and comparing them to the experimental FRET histograms. Other methods such as lifetime-filtered fluorescence correlation spectroscopy, two-dimensional lifetime correlation spectroscopy, and RASP, first process the photon arrival times by computing correlation functions, frequency domain maps, or FRET histograms at different delay times. The preprocessed data are then used for model fitting. As an advantage, the presence of dynamics can already be inferred from the preprocessed data by eye, thus simplifying a model guess. On the other hand, these methods often require long measurements to obtain a high signal/noise in the processed data.

Not standardly accounted for in these methods is static heterogeneity due to dye isomers or permutations of donor and acceptor positions. The latter is particularly prevalent in smFRET as donor and acceptor labeling is often done at cysteine residues, thus resulting in a mixture of labeling permutations. Burst variance analysis (BVA) (27) is a popular tool to identify both static and dynamic heterogeneity. Yet, BVA has mainly been used as a qualitative indicator for dynamics (3) as kinetic rates remain inaccessible. Here, we present an extension of BVA (27) termed time-resolved BVA (trBVA) that is also able to quantify kinetic rates from smFRET experiments of freely diffusing molecules between 200 ms^{-1} (5 μs) and 0.2 ms^{-1} (5 ms) with an error of a factor of 1.5. The method does not require long measurements and is easy to implement. To benchmark the robustness of trBVA, we performed smFRET simulations of dynamic particles and also applied the method to real single-molecule data of labeled DNA and protein. We hope that trBVA will be a useful extension of the current smFRET analysis toolbox to identify biomolecular dynamics at timescales from micro- to milliseconds.

MATERIALS AND METHODS

Theory

A photon burst i from a biomolecule labeled with donor (D) and acceptor (A) that diffuses through the confocal volume of a microscope contains d_i donor and a_i acceptor photons. The total number of detected photons in the burst is $n_i = a_i + d_i$ (including background photons), and the total number of bursts is N. We denote the uncorrected FRET efficiency as ϵ and the corrected FRET efficiency as E (corrected for the differences in quantum yield of the dyes, cross talk between channels, background, and acceptor direct excitation; see section burst identification and data preprocessing). The idea of classical BVA is to partition photons of a burst into segments of m (typically $m = 5$) consecutive photons. For each of these $M_i = \lfloor n_i/m \rfloor$ photon segments, the uncorrected FRET efficiency ϵ_{ij} (segment index j) is computed. Finally, we then calculate the variance of ϵ using all segments of the N bursts

$$s^2 = \frac{1}{\left(\sum_{i=1}^{N} M_i\right) - 1} \sum_{i=1}^{N} \sum_{j=1}^{M_i} \left(\epsilon_{ij} - \langle \epsilon \rangle\right)^2$$

$$\text{with } \langle \epsilon \rangle = \frac{1}{\sum_{i=1}^{N} M_i} \sum_{i=1}^{N} \sum_{j=1}^{M_i} \epsilon_{ij}$$

$$= \sum_{i=1}^{N} a_i \Big/ \sum_{i=1}^{N} n_i. \qquad (1)$$

The expected FRET variance of these segments in the absence of both dynamic and static heterogeneity (notably, Eq. 2 is also correct in the limit at which multiple states interconvert at timescales faster than the interphoton time), i.e., assuming the presence of only a single state, is due only to shot noise, and is given by

$$\sigma^2 = \frac{\langle \epsilon \rangle (1 - \langle \epsilon \rangle)}{m}. \qquad (2)$$

The excess variance due to conformational heterogeneity is then given by the difference between Eqs. 1 and 2:

$$S^2 = s^2 - \sigma^2. \qquad (3)$$

Importantly, the analysis can also be performed with a subset of the N bursts. For instance, in a FRET-resolved trBVA version, the excess variance (Eq. 3) is computed for a set of bursts that lie within a chosen FRET efficiency range. If $S^2 > 0$, the FRET variance exceeds the shot noise expectation, thus indicating static or dynamic heterogeneity. The basic idea of trBVA is to vary the length m of the photon segments (Fig. 1 *A* and *B*). Clearly, both variances s^2 and σ^2 will change with m, but these changes will not be identical such that S^2 is itself a function of m. This function therefore contains information about the heterogeneity among and within bursts, which either is static or dynamic, i.e., time dependent. To extract this information, we derived an analytical expression for the excess variance of the subset of m-photon segments with specific time duration t, which we call the "t-specific excess variance" (Appendix I). Here, t is defined as the length of the time interval between the first and the last photon of a segment. Writing the FRET autocorrelation function as $g(t) = \langle \delta\epsilon(0)\delta\epsilon(t) \rangle$ with $\delta\epsilon(t) = \epsilon(t) - \langle \epsilon \rangle$, we obtain

$$\Delta s^2(m,t) = \frac{1}{m^2}\left[2\,g(t) + \frac{4(m-2)}{t}\int_0^t g(t')dt' + \frac{2(m-2)(m-3)}{t^2}\int_0^t (t-t')g(t')dt'\right] \qquad (4)$$

Importantly, for the ensemble of all m-photon segments, the time window t is a random variable with a conditional probability density

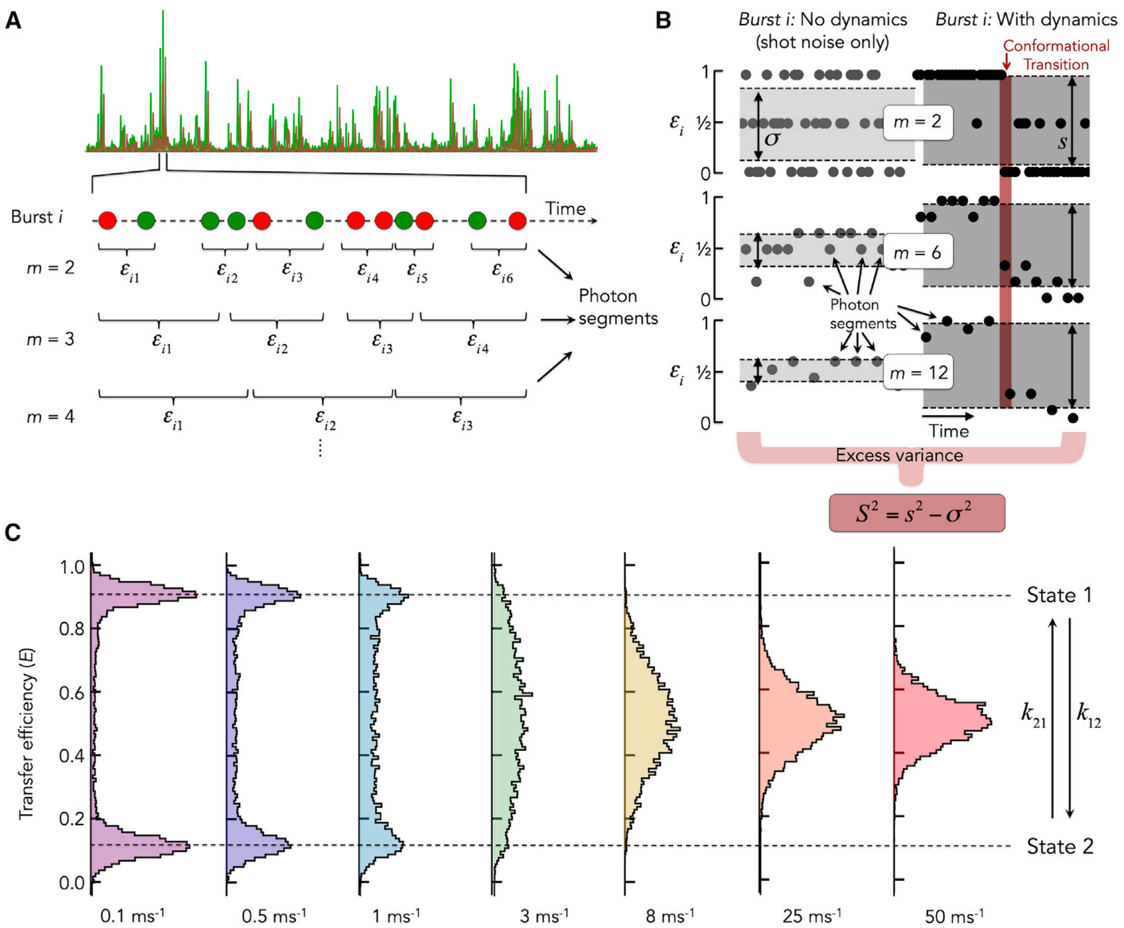

FIGURE 1 Scheme of the trBVA procedure and simulated FRET efficiency histograms (corrected) for a freely diffusing dynamic particle. (*A*) In trBVA, the photons from acceptor (*red*) and donor (*green*) in a burst *i* are partitioned into segments of length *m*. For each segment, apparent (uncorrected) FRET values ϵ_{ij} are computed, and the variance of these FRET values is studied as function of *m*. (*B*) Illustration of the trBVA variance analysis. The FRET efficiencies of individual segments within a burst *i* are depicted as function of time for two scenarios: a hypothetical burst without dynamics, i.e., only including shot noise (*left column*), and a "measured" burst with a conformational transition (*right column*). The FRET efficiencies are shown for three values of segment lengths *m* (indicated). The variances of FRET efficiencies are depicted as gray shaded areas. The trBVA excess variance is the difference between the measured variance (*right*) and the shot noise variance (*left*). Importantly, trBVA excess variance for a given segment length *m* is computed from the segments of all bursts, not only for a single burst as shown in *B*. (*C*) Brownian dynamics simulation of FRET efficiency histograms (corrected) for a particle diffusing freely through a confocal spot including bleaching of donor and acceptor. The particles switched between two states with corrected FRET efficiencies $E_1 = 0.9$ and $E_2 = 0.1$. The kinetic forward (k_{12}) and backward (k_{21}) rates were assumed to be identical. The FRET efficiency histograms are shown for different values of k_{12} and k_{21} (*bottom*).

function $P(t|m)$. Once $P(t|m)$ is known, the excess variance due to conformational dynamics as function of *m* can be calculated from the following:

$$S^2(m) = \int_0^\infty P(t|m)\Delta s^2(m,t)dt. \quad (5)$$

The change of S^2 with increasing *m* can therefore be computed by knowing the autocorrelation function $\langle \delta\epsilon(0)\delta\epsilon(t)\rangle$ and the distribution $P(t|m)$. The autocorrelation function can be easily computed for any kinetic model. If $\mathbf{K}$ is the rate matrix of the model, $\mathbf{p}_{eq}$ is the population vector of conformational states at equilibrium ($\mathbf{K}\mathbf{p}_{eq} = 0$), and ϵ is a diagonal matrix with the same dimensions as $\mathbf{K}$ whose diagonal elements are the FRET efficiencies of each conformational state, then the FRET autocorrelation function can be expressed as (8)

$$g(t) = \mathbf{1}^{\mathrm{T}}\epsilon e^{\mathbf{K}\,t}\epsilon\,\mathbf{p}_{eq} - \left(\mathbf{1}^{\mathrm{T}}\epsilon\,\mathbf{p}_{eq}\right)^2, \quad (6)$$

where $\mathbf{1}$ is a vector of ones. For instance, a model, in which two states with FRET efficiencies ϵ_1 and ϵ_2 interconvert with rates k_{12} and k_{21}, has the correlation function

$$g(t) = \langle\delta\epsilon^2\rangle e^{-(k_{12}+k_{21})t} \text{ with } \langle\delta\epsilon^2\rangle = \frac{k_{12}k_{21}}{(k_{12}+k_{21})^2}(\epsilon_2 - \epsilon_1)^2. \quad (7)$$

A fit of S^2 with Eqs. 4, 5, and 7 would provide the two unknown quantities $\langle\delta\epsilon^2\rangle$ and $k_{obs} = k_{12} + k_{21}$ if $P(t|m)$ was known. In fact, this distribution can be extracted from the experimental data

directly. We first determine the time duration of all photon segments of length m for all bursts or a subset of bursts within a chosen FRET window $E_l \le E < E_l + \Delta E$ in the FRET-resolved version. A histogram of these times $H(t_i|m)$ for equally spaced time bins t_i with $i = \{1, 2, 3, ..., K\}$ then provides a reasonable estimate for $P(t|m)$. For data fitting, we therefore use Eq. 5 in discrete form:

$$S^2 = \sum_{i=1}^{K} H(t_i|m)\Delta s^2(m, t_i) \Big/ \sum_{i=1}^{K} H(t_i|m). \quad (8)$$

For completeness, we also provide the explicit forms of $\Delta s^2(m, t)$ for a two-state and a three-state system in Appendix II. For comparison, we also computed the donor-acceptor cross correlation function $G_{DA}(\tau) = \langle n_D(t')n_A(t'+\tau)\rangle / \langle n_D\rangle\langle n_A\rangle$ for the selected bursts. Here, $n_D(t')$ and $n_A(t')$ are the photon counts at time t'. To extract the relaxation time, $G_{DA}(\tau)$ was fitted with the empirical function

$$f(\tau) = a\left(1 - e^{-k_{obs}\tau}\right) + be^{-(\tau/t_D)^{\beta}} + c. \quad (9)$$

Here, $k_{obs} = k_{12} + k_{21}$ is the observed rate of conformational changes, t_D is an empirical timescale to describe the decay of $G_{DA}(\tau)$ due to diffusion, and β is a stretching exponent.

Data simulation

To test the accuracy of trBVA in extracting kinetic rates from smFRET experiments, we simulated photon time traces of diffusing particles that switch between two conformational states (1 and 2) described by kinetic rate coefficients k_{12} and k_{21}. The FRET efficiencies of the two states were $E_1 = 0.1$ and $E_2 = 0.9$, respectively. The diffusion of the particle through the confocal volume was modeled via Brownian dynamics simulations with the software package Fretica (https://schuler.bioc.uzh.ch/programs/), developed by Daniel Nettels and Benjamin Schuler (University of Zurich). The Stokes radius of the particles was set to 4.3 nm, which corresponds to a medium-sized protein, and the particles diffused in a solvent with the viscosity of water at 25°C, i.e., 1 mPas, resulting in a diffusion coefficient of $5 \times 10^{-5}\ \mu\text{m}^2/\mu\text{s}$. The simulation was initialized by randomly placing particles in a simulation sphere with a radius of $R = 3\ \mu\text{m}$. The number of initial particles was drawn from a Poisson distribution with a mean $n_0 = \frac{4}{3}\pi R c_0$ with a bulk particle concentration of $c_0 = 50\ \text{pM}$. The simulation was performed in spherical coordinates assuming for simplicity radial symmetry of the confocal volume, which is located at the origin. Brownian motion is simulated using the following:

$$r(t + \Delta t) = r(t) + \frac{2D\Delta t}{r(t)} + \Delta r. \quad (10)$$

Here, $r(t)$ is the radial distance at simulation steps $t = 1...T$, where T is the length of the simulation in steps of $\Delta t = 1\ \mu\text{s}$, i.e., the time between two simulation steps, D is the diffusion coefficient, and Δr is a random distance drawn from a normal distribution with zero mean and a variance $\sigma_{\Delta r}^2 = 2D\Delta t$. Each particle is simulated until it leaves the simulation sphere. To ensure a constant mean concentration of particles near the center of the sphere, the particle loss at the sphere's surface is compensated by periodically (periodicity T_{new}) placing new particles inside the sphere near the boundary. The distribution of new particles $c_{new}(r)$ that entered the sphere after time T_{new} is obtained by solving the radial diffusion equation

$$\frac{\partial c}{\partial t} = D\left(\frac{\partial^2 c}{\partial r^2} + \frac{2}{r}\frac{\partial c}{\partial r}\right) \quad (11)$$

with the initial condition $c(r < R, t = 0) = 0$ and the boundary conditions $c(r = R, t) = c_0$ and $c(r \to 0, t) = 0$. The solution is known (28) and given by

$$\frac{c(r,t)}{c_0} = 1 + \frac{2R}{\pi r}\sum_{n=1}^{\infty}\frac{(-1)^n}{n} sin\left(\frac{n\pi r}{R}\right) exp\left(-Dn^2\pi^2 t / R^2\right) \text{with } c_{new}(r) = c(r, T_{new}). \quad (12)$$

The mean number of new particles entering the sphere is then computed by integrating over the volume of the sphere:

$$\frac{n_{new}}{n_0} = 1 + \frac{6}{\pi^2}\sum_{n=1}^{\infty}\frac{1}{n^2} exp\left(-Dn^2\pi^2 T_{new} / R^2\right). \quad (13)$$

After each time interval T_{new}, a random number of new particles was drawn from the Poisson distribution with mean n_{new}. The particles were placed at radial distances randomly chosen from the distribution with the density function $P_{new}(r) = 4\pi r^2 c_{new}(r)/n_{new}$ for $r < R$. In total, we simulated particle trajectories for 1800 s. Once the particle trajectories were simulated, we added conformational dynamics simulated according to the rate equation

$$\frac{d\mathbf{p}}{dt} = \mathbf{Kp}, \quad (14)$$

where $\mathbf{p}$ is the population vector of four states: low FRET (DA_1) with FRET efficiency E_1, high FRET (DA_2) with FRET efficiency E_2, donor-only (D), and acceptor-only (A) in the basis $\{D, DA_1, DA_2, A\}$. The rate matrix $\mathbf{K}$ is a combination of the rate matrix $\mathbf{K}_0$ for conformational transitions between DA_1 and DA_2 and the rate matrix $\mathbf{K}_{bl}$ describing photophysical effects, photobleaching in our case,

$$\mathbf{K} = \mathbf{K}_0 + I(r)\mathbf{K}_{bl} \text{ with} \quad (15)$$

$$\mathbf{K}_0 = \begin{pmatrix} 0 & 0 & 0 & 0 \\ 0 & -k_{12} & k_{21} & 0 \\ 0 & k_{12} & -k_{21} & 0 \\ 0 & 0 & 0 & 0 \end{pmatrix} \quad (16)$$

$$\mathbf{K}_{bl} = \begin{pmatrix} 0 & k_a E_1 & k_a E_2 & 0 \\ 0 & -k_a E_1 - k_d(1 - E_1) & 0 & 0 \\ 0 & 0 & -k_a E_2 - k_d(1 - E_2) & 0 \\ 0 & k_d(1 - E_1) & k_d(1 - E_2) & 0 \end{pmatrix} \quad (17)$$

with the bleaching rates k_a and k_d for acceptor and donor fluorophores located at the origin ($r = 0$), respectively. We assumed a bleaching timescale of $k_a = k_d = 5 \times 10^{-4}\ \mu s^{-1}$ for the simulations. The position-dependent profile $I(r)$ that accounts for the illumination intensity at different positions in the confocal volume is given by

$$I(r) = exp\left(-\frac{2r^2}{w_0^2}\right) \text{ with } w_0 = 0.4\ \mu\text{m}. \quad (18)$$

For each particle with the diffusion trajectory $r(t)$ and starting time t_0, a random state trajectory s_t is simulated according to Eqs. 14–18 with the program Fretica. The initial state $s(t_0)$ was chosen randomly according to the initial probabilities for the four states given by the vector $\mathbf{p}_0$ with the same basis as $\mathbf{p}$. We chose an equal distribution of high- and low-FRET species and the same number of donor-only and acceptor-only molecules with $\mathbf{p}_0 = \left(0.1, \quad 0.8\frac{k_{21}}{k_{12}+k_{21}}, \quad 0.8\frac{k_{12}}{k_{12}+k_{21}}, \quad 0.1\right)^T$. In addition, we set the total photon rate at the center of the excitation volume to $\lambda_{tot} = 0.4\ \mu s^{-1}$ and introduced realistic background photon rates of $\lambda_d = 5.6\ 10^{-3}\ \mu s^{-1}$ for the donor channel and $\lambda_a = 3\ 10^{-3}\ \mu s^{-1}$ for the acceptor channel. To model the experimental situation in a realistic fashion, we also introduced different detection efficiencies for the dyes ($\gamma = Q_a\eta_a/Q_d\eta_d = 1.16$), where $Q_{a,d}$ and $\eta_{a,d}$ are the quantum yields and detection efficiencies for acceptor and donor dye, cross talk (leakage) between donor photons in the acceptor channel ($\beta = 0.054$), and the probability to directly excite the acceptor with the donor excitation laser ($\alpha = 0.048$). As we introduced donor-only and acceptor-only molecules together with the possibility of photobleaching, we also simulated pulsed-interleaved excitation (PIE) of both dyes with $\gamma_{PIE} = 2$ (6,29). To this end, experimental instrumental response functions were used to generate the photon distributions after donor and acceptor excitation within one PIE period. Finally, a time-tagged time-resolved file containing the simulated photons was generated. Simulations of a three-state model were performed in the same manner.

Burst identification and data preprocessing

After simulating photon traces based on the kinetic model described above, the time-tagged time-resolved file was processed with standard single-molecule analysis tools (6) for generating corrected FRET efficiency histograms. Importantly, for the calculation of variances for BVA, raw photon counts, without correction, were used to calculate apparent FRET efficiencies, also known as proximity ratios. Unless stated otherwise, the photon trajectory was binned into time windows of 100 µs. A burst is defined as a collection of consecutive bins with more than two photons per bin and a total photon number of at least 100 photons after donor excitation. The corrections included background, differences in the brightness of donor and acceptor, channel cross talk, and acceptor direct excitation. The procedure is described in detail elsewhere (6,30). The corrected photon numbers of donor (n_{DD}) and acceptor (n_{DA}) after donor excitation were used to compute the FRET efficiency of the burst via

$$E = \frac{n_{DA}}{n_{DA} + n_{DD}}. \quad (19)$$

To exclusively identify molecules that contain both dyes, we computed the stoichiometry ratio for each burst via

$$S_{PIE} = \frac{n_{DD} + n_{DA}}{n_{DD} + n_{DA} + \gamma_{PIE} n_{AA}}. \quad (20)$$

Only bursts with $S_{PIE} < 0.65$ were retained for further analysis. Since bursts were identified based on photon counts after donor excitation, molecules without donor were automatically excluded from the analysis. To also exclude bursts in which the acceptor bleached during the transit of the particle through the confocal volume, we further selected bursts in which the mean detection time of photons was similar after donor and acceptor excitation. We define

$$\alpha_{PIE} = \langle t_{Dex} \rangle - \langle t_{Aex} \rangle, \quad (21)$$

where $\langle t_{Dex} \rangle$ and $\langle t_{Aex} \rangle$ are the mean detection times (in ms) after donor and acceptor excitation, respectively. Including shot noise, the asymmetry value α_{PIE} has a standard deviation given by

$$\sigma_{PIE} = \frac{T}{2\sqrt{3}}\sqrt{\frac{1}{n'_{DD} + n'_{DA}} + \frac{1}{n'_{AA}}} \quad (22)$$

where the prime indicates the uncorrected photon counts. We chose a restrictive threshold of $\sigma_{PIE} < 0.15$ to exclude bursts with bleached acceptors.

RESULTS

Global trBVA

To test the ability of trBVA (Fig. 1 *A*) in quantifying timescales of conformational dynamics, we simulated the photon emission process for freely diffusing molecules in a photon-by-photon manner. We modeled molecules that switch between two conformational states 1 and 2 with "forward" rate k_{12} and "backward" rate k_{21}. The corrected FRET efficiencies (E) of the two states were $E_1 = 0.1$ and $E_2 = 0.9$, which corresponds to the uncorrected values $\epsilon_1 \approx 0.2$ and $\epsilon_2 \approx 0.9$. For simplicity, we assumed identical rates in both directions. At a slow exchange rate of $k_{12} = k_{21} = 0.1\ \text{ms}^{-1}$, i.e., one transition per ten milliseconds on average, the FRET efficiency histogram shows two well-separated peaks with shot noise limited width at the expected FRET efficiencies (Fig. 1 C). Intermediate values between the dominant peaks become prominent with increasing exchange rates, as more molecules change their conformation while diffusing through the confocal volume. At higher rates, the FRET peaks start to coalesce, and at the highest exchange rate of $k_{12} = k_{21} = 50\ \text{ms}^{-1}$, the FRET peaks merged completely, thus giving the impression of a single conformational state. To analyze these data with trBVA, we computed the variance of FRET fluctuations by partitioning bursts into consecutive segments with m photons (Fig. 1 *A*). As outlined in the theory section, computing the variance of these segments and subtracting the shot noise contribution one would have if there was a single state with a FRET efficiency equal to the population weighted mean of the states, provides the excess variance S^2 (Eqs. 1–3). Fig. 2 *A* demonstrates that S^2 first increases and then decreases with increasing size of

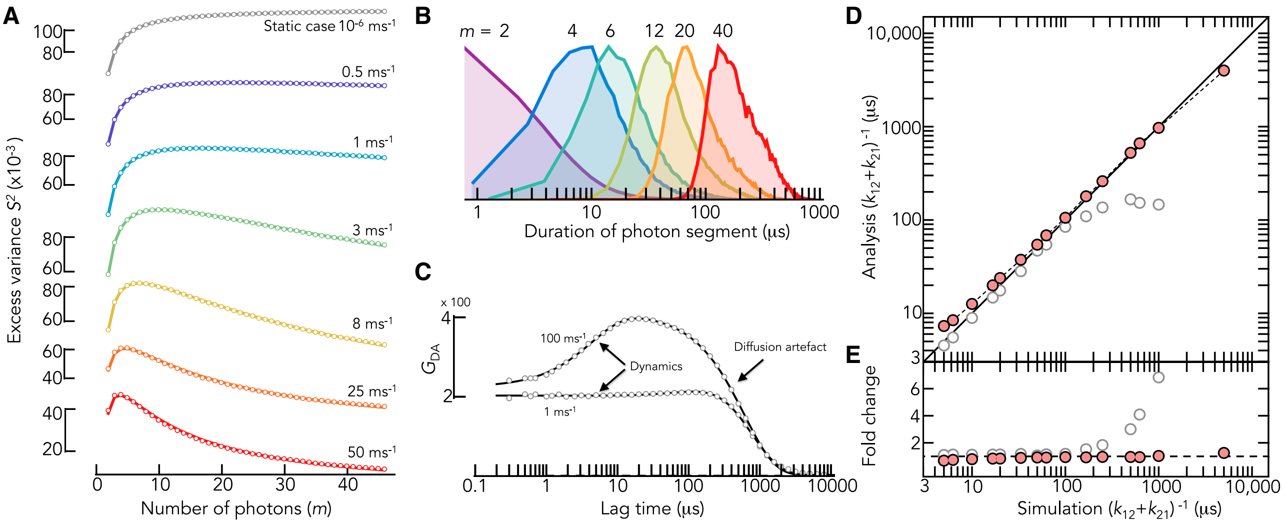

FIGURE 2 Kinetic analysis with trBVA. (*A*) Traces of the excess variance S^2 with increasing number m in the photon segments for different values of $k_{12} = k_{21}$ (indicated). Solid lines are fits with Eqs. 4, 7, and 8. The fits had two fitting parameters, the amplitude $\langle \delta\epsilon^2 \rangle$ and the observed rate $k_{obs} = k_{12} + k_{21}$. (*B*) Distribution of the duration of the photon segments $H(t_i|m)$ for different photon numbers (indicated). (*C*) Donor-acceptor cross correlation functions for two exchange rates (indicated). The solid line is a fit to Eq. 9. (*D*) Comparison of the relaxation times $(k_{12} + k_{21})^{-1}$ between simulation (ground truth) and extracted from trBVA (*red circles*). Gray circles are relaxation times obtained from the cross correlation functions (see *C*). Solid line is the identity line. (*E*) Ratio of the relaxation times between simulation and the analysis with trBVA (*red circles*) and the analysis of the cross correlation (*gray circles*).

the photon segments m. The trBVA traces obtained from the data (Fig. 2 *A*) can now be used to determine the apparent relaxation time $\tau = (k_{12} + k_{21})^{-1}$ of the conformational fluctuations using the experimentally determined distribution $H(t_i|m)$ of the time duration of m-photon segments. Examples are shown in Fig. 2 *B*. For $m = 2$, the distribution is a decaying function as expected based on photon counting theory (8). For higher values of m, $H(t_i|m)$ shows a clear maximum due to the fact that a successive emission of several photons causes a delay between the first and the mth photon that leads to the rise at short times. To fit the trBVA traces, we use Eqs. 4, 7, and 8 to compute S^2 for each value of m and minimize the least squares difference $\chi^2 = \sum_m [S^2_{experiment}(m) - S^2_{fit}(m)]^2$. The fit contains two parameters, the amplitude of the FRET correlation function $\langle \delta\epsilon^2 \rangle$ and the kinetic rate $k_{obs} = k_{12} + k_{21}$ (Eq. 7), i.e., the eigenvalue of the rate matrix. The fits provide an excellent description of the simulated data over a broad range of exchange rates (Fig. 2 *A*). An alternative method to determine kinetic rates would be to compute the FRET autocorrelation function directly or analogously, the donor-acceptor cross correlation $G_{DA}(\tau) = \langle n_A(t) n_D(t+\tau) \rangle / \langle n_A \rangle \langle n_D \rangle$ for the data (Fig. 2 *C*). Distance dynamics lead to a rise of the cross correlation amplitude since donor and acceptor signal are anticorrelated. Yet, the finite burst duration causes an additional decay in $G_{DA}(\tau)$ at the timescale at which molecules diffuse through the confocal spot. This diffusion amplitude dominates $G_{DA}(\tau)$, and slow dynamics at timescales close to the diffusion are difficult to identify (Fig. 2 *C*). This problem is circumvented with trBVA.

A comparison of the apparent relaxation times $\tau = (k_{12} + k_{21})^{-1}$ from the trBVA analysis with the true values used in the simulation demonstrates an excellent agreement (Fig. 2 *D*). For dynamics across three orders of magnitude (5 µs to 5 ms), trBVA provides estimates of τ with less than twofold deviation from the ground truth (Fig. 2 *E*). Even dynamics slower than the diffusion of molecules through the confocal spot can be obtained. The reason for this surprising result is that S^2 is bounded by two limits. For dynamics much faster than the experimental interphoton time, the lower boundary is given by $S^2(m) = 0$ (Appendix III). Yet, for extremely slow dynamics, the FRET autocorrelation function is approximately constant ($g(t) \approx \langle \delta\epsilon^2 \rangle$) but different from zero. Under this condition, the excess variance is given by $S^2 = \langle \delta\epsilon^2 \rangle (1 - m^{-1})$, which is an increasing function of m and represents the upper boundary (Fig. 2 *A*, top). Notably, this increase is not in conflict with the central limit theorem. The total variance indeed decreases with increasing m (Appendix III). Instead, the increase of S^2 results from an inaccurate estimate of shot noise (Eq. 2) in the presence of static heterogeneity (Appendix III). Importantly, even slight deviations from the $(1 - m^{-1})$ dependence require a finite decay time in

$g(t)$, which explains the success of trBVA at slow timescales. Notably, this is a helpful feature to identify static heterogeneity. For instance, labeling proteins with donor and acceptor is often done via two cysteine residues, which unavoidably results in two labeling permutations. If the molecular brightness of the dyes differs in the two variants, they will exhibit different FRET efficiencies, and S^2 will follow the $(1 - m^{-1})$ dependence. In comparison to trBVA, the relaxation times from the donor-acceptor cross correlation function $G_{DA}(\tau)$ are highly inaccurate at the diffusion timescales (Fig. 2 *D*). Compared to the 1.5-fold error in trBVA, the cross correlation analysis deviates from the ground truth sevenfold at a relaxation time of 1 ms.

FRET-resolved trBVA

Similar to regular BVA, also the time-resolved version can be used to investigate dynamics in different regions of the FRET efficiency histogram. In FRET-resolved trBVA, the segments of bursts within a particular FRET range are analyzed. Importantly, selecting bursts within a FRET range means selecting trajectories according to their mean FRET efficiency. In a two-state system, S^2 for bursts with FRET values different from the ensemble average will therefore be biased. Bursts with FRET values substantially lower than the ensemble average will contain trajectories with longer dwell times in the low-FRET state and shorter dwell times in the high-FRET state (Fig. 3 *A*). The opposite happens when bursts with substantially higher FRET than the ensemble average are being selected. As the observed rate is a sum of forward and backward rate, the faster rate, i.e., the shorter dwell time, dominates. Hence, at the flanks of the FRET efficiency distribution, the observed exchange rates will in general be higher than the correct value (Fig. 3 *B*, top). The steep change of the rate at the flanks of the distribution is therefore indicative of leaving the FRET regime in which dynamics occur. Similar information is contained in the amplitude $\langle \delta\epsilon^2 \rangle$ of the FRET autocorrelation function. For a two-state system, the populations of both states in a trajectory

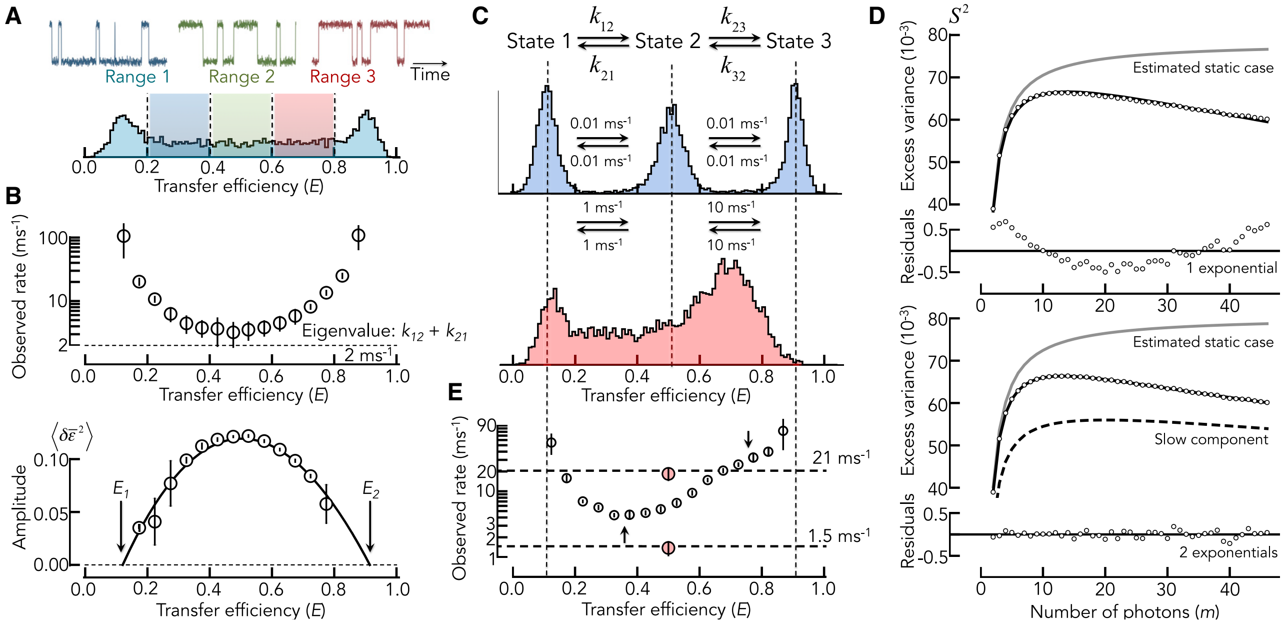

FIGURE 3 Benefits and pitfalls of FRET-resolved trBVA. (*A*) FRET histogram of a two-state system interconverting at a rate of 1 ms^{-1} (same as in Fig. 1). Three FRET ranges are indicated. Idealized schematics of trajectories that might be found in the three ranges (*top*). (*B*) Exchange rate as function of FRET for the data shown in (*A*) obtained with single-exponential correlation functions (*top*). The true rate (eigenvalue) is indicated as dashed line. Amplitudes $\langle \delta\epsilon^2 \rangle$ of the FRET autocorrelation function as a function of corrected FRET efficiency for the data in (*A*) (*bottom*). The solid line is a fit with the second-order polynomial Eq. 23. Error bars are from five independent simulations. (*C*) Model of a three-state system (*top*) and simulated FRET histograms without (*middle*) and with (*bottom*) fast exchange. The forward and backward rates for the individual reactions are indicated. (*D*) Decays of the excess variance as function of *m* and fits with Eqs. II.3 and 8 (*solid black lines*) for a two-state model with a single-exponential correlation function (*top*) and a three-state model (Eqs. II.4 and 8) with a double-exponential correlation function (*bottom*). The dashed line indicates the component with the low eigenvalue. Gray lines indicate the static case estimated from the fit obtained by setting all eigenvalues to zero. (*E*) Observed exchange rate obtained with single-exponential fits as function of the corrected FRET efficiency for the three-state system (*white circles*). Horizontal dashed lines indicate the true eigenvalues of the system, and the arrows highlight regimes of exchange. The rates obtained from a fit of all bursts with a three-state model (see *D*, *bottom*) are shown as red circles. Error bars are from five independent simulations.

with an arbitrarily chosen uncorrected FRET value $\bar{\epsilon}$ are given by $p_1' = (\epsilon_2 - \bar{\epsilon})/(\epsilon_2 - \epsilon_1)$ and $p_2' = (\bar{\epsilon} - \epsilon_1)/(\epsilon_2 - \epsilon_1)$, respectively. The primes indicate that these occupancies differ from those of the whole ensemble of molecules. The amplitude of the FRET autocorrelation at this FRET value is $\langle \delta\bar{\epsilon}^2 \rangle = p_1' p_2' (\epsilon_2 - \epsilon_1)^2$ (see also Eq. 7), which can be re-written as

$$\langle \delta\bar{\epsilon}^2 \rangle = -(\bar{\epsilon} - \epsilon_1)(\bar{\epsilon} - \epsilon_2). \tag{23}$$

Hence, the amplitude follows a second-order polynomial in $\bar{\epsilon}$ where the roots identify the position of the states (Fig. 3 *B*). Notably, this relationship is independent of the true relative populations of the two states (p_1 and p_2). The amplitude analysis is therefore suited to identify the (uncorrected) FRET values of the interconverting states ϵ_1 and ϵ_2. In general, FRET-resolved trBVA experiments can be used to identify the positions of FRET states. However, kinetic rates should always be inferred from S^2 using all bursts and not from FRET-resolved trBVA! This is important as the FRET-dependent rates will always exhibit a minimum at a FRET value centered between ϵ_2 and ϵ_1, i.e., the point at which $p_1' = p_2'$, irrespective of the abundance of both conformers in the whole ensemble. Moreover, the observed rate at the minimum is higher than the eigenvalue of the system (Fig. 3 *B*, top) because trajectories without transitions (bursts with ϵ_1 and ϵ_2) are underrepresented in this FRET range. To exemplify this deviation, we simulated a more complicated system in which three states with different FRET efficiencies ($E_1 = 0.1$, $E_2 = 0.5$, $E_3 = 0.9$) interconvert at different timescales (Fig. 3 *C*). We assume that state 1 and 2 exchange at a slow timescale with the rates $k_{12} = k_{21} = 1\ ms^{-1}$, whereas state 2 and 3 exchange an order of magnitude faster with $k_{23} = k_{32} = 10\ ms^{-1}$. A comparison with the case in which exchange is hundredfold slower than the diffusion time through the detection volume shows how drastically dynamics can alter the appearance of FRET efficiency distributions (Fig. 3 *C*). In the presence of fast exchange at two different timescales, the FRET efficiency histogram shows a major peak at an apparent FRET efficiency value of 0.7, a minor peak at 0.1, and a floor of events in between the peaks. In a quantitative global analysis, we first computed S^2 for all bursts. As expected, the trBVA trace increases and decreases with *m* (Fig. 3 *D*). A fit with a single-exponential FRET correlation function (Eqs. 4, 7, and 8) already provides a reasonable fit (Fig. 3 *D*, top). Yet, the residuals clearly show discrepancies between data and fit. Indeed, a fit with a double-exponential correlation function, which corresponds to the correct three-state model (Appendix II), provides an excellent description of the data (Fig. 3 *D*, bottom) and gives the correct eigenvalues (Fig. 3 *E*). To exemplify how static heterogeneity would manifest in trBVA, we set the fitted rates in the correlation functions to zero (Fig. 3 *D*). The comparison shows that dynamics lower the amplitude of the trBVA trace and introduces the decay at large *m*. In the more qualitative FRET-resolved rate analysis, we calculated trBVA traces for bursts with different FRET efficiency values. An empirical fit with a single-exponential FRET correlation function provides apparent exchange rates for the individual FRET efficiency values. These rates exhibit a nontrivial FRET dependence (Fig. 3 *E*). A minimum is observed at FRET values between state 1 and 2. Starting from the minimum, the exchange rates increase toward lower FRET values as expected (compare to Fig. 3 *B*, top). However, although the rates also increase toward higher FRET values, a flattening of this dependence between state 2 and 3 is found. The position coincides with the position of the major peak at high FRET, which can be taken as indication that molecules in this peak dynamically switch at a fast timescale. Yet, the analysis is qualitative as the rates at both minimum and flattening point are substantially higher than the eigenvalues (Fig. 3 *E*).

As a rule of thumb, steep changes in exchange rates along the FRET coordinate indicate regions with biased trajectories and therefore regions close to the positions of the FRET states. FRET-independent exchange rates (minima or flat regions in the rate profile) indicate trajectories with strong exchange between states. Yet, care has to be taken as 1) flattening of the rate profile might not always be clearly visible, and 2) states in exchange rarely have identical populations such that exchange rates should not be inferred from the rate-FRET profile but always from the trBVA decay of the whole ensemble.

Probing the dynamics of double-stranded DNA

As an application of trBVA, we probed the dynamics of double-stranded DNA (dsDNA) breathing. Structural fluctuations in dsDNA have previously been measured using fluorescence quenching (31). A relaxation time of $\sim$50 μs was found for these local opening-closing motions, a timescale well within the regime that can be probed with trBVA. We performed smFRET experiments on dsDNA at neutral and acidic pH. At acidic pH, dsDNA is known to be destabilized (32) due to the protonation of DNA bases, and we expect a significant difference in the amplitude and/or timescales of these motions between pH 7 and pH 4. We generated 12 dsDNA samples of 84 bp length each that were derived from a naturally occurring promoter sequence in *Bacillus subtilis* (33). The samples were

site-specifically labeled with AlexaFluor488 as donor and AlexaFluor594 as acceptor at varying positions, thus spanning the full FRET efficiency range from low to high values. We performed short 5- to 10-min-long experiments using PIE (29) and identified bursts as described in the materials and methods section. As expected, the FRET efficiency histograms of these samples span the full FRET range (Fig. 4 *A*). Notably, the widths of the FRET efficiency histograms are significantly increased at pH 4 compared to pH 7, suggesting that the drop in pH either alters the timescales of distance dynamics or the amplitude or both (Fig. 4 *A*). We then used trBVA to analyze FRET fluctuations in these samples. A comparison of S^2 at $m = 5$ shows a substantially increased fluctuation amplitude at pH 4 compared to pH 7 (Fig. 4 *A*). This variance is reduced at $m = 46$, suggesting a pronounced microsecond decay. An overview of the decays indeed demonstrates the presence of structural fluctuations that are intensified at low pH (Fig. 4 *B*). A fit with a two-state model provides an empirical description of these data with relaxation times that are in rough accord with the previous estimate of 50 μs at dsDNA samples with intermediate FRET efficiencies, whereas substantially larger relaxation times were found for samples with extremely low and high FRET values. However, the fits do not properly capture the trBVA decays. To obtain a better description of the traces, we also fitted with double-exponential FRET autocorrelation functions, which is equivalent to a model with three states. This model describes all trBVA traces well and results in two relaxation times (Fig. 4 *C*). The fast relaxation time is closest to the previous estimate of 50 μs at samples with low FRET efficiencies (Fig. 4 *C*, bottom).

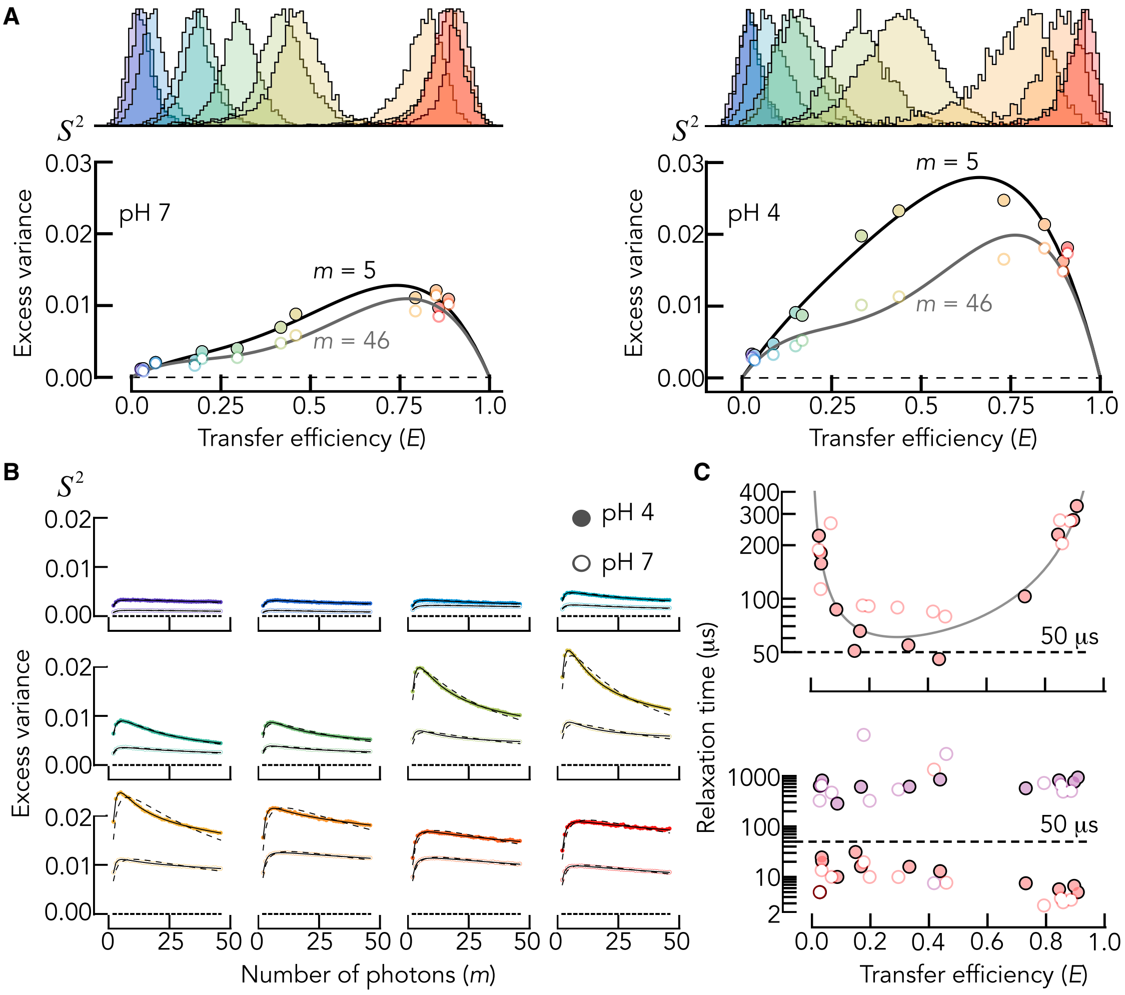

FIGURE 4 Probing dsDNA breathing motions. (*A*) FRET histograms (*top*) and trBVA amplitudes (*bottom*) for dsDNA samples at pH 7 (left) and pH 4 (*right*). Solid lines are fits to a fourth-order polynomial. (*B*) TrBVA traces and fits of all dsDNA samples with Eqs. 4, 6, and 8. Using a FRET autocorrelation function with one (*dashed*, Eq. II.3) and two (*solid*, Eq. II.4) exponentials. Colors are identical to (*A*). (*C*) Relaxation rates of dsDNA motions at pH 7 (open circles) and pH 4 (*solid circles*) for the fits with one (*top*) and two (*bottom*) exponentials. A relaxation time of 50 μs is indicated.

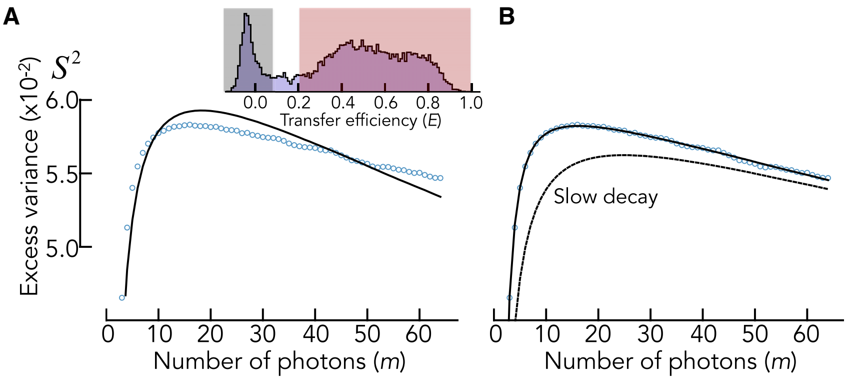

FIGURE 5 Probing the folding and unfolding of a protein. (*A*) TrBVA decay of BDPA (*circles*) and a fit with a two-state model (*solid line*). Inset: FRET efficiency histograms of BDPA. Red shaded area highlights bursts used for trBVA. The gray area indicates molecules without an active acceptor. (*B*) Same data as in (*A*) with a fit of a three-state model (*solid line*). The dashed line shows the contribution from the slow decay component with a rate of 0.9 ms^{-1} and a relative amplitude of 64%.

Yet, for samples with high FRET efficiency, the fast relaxation time drops to values in the order of 2–10 μs. This very fast timescale could be caused by transitions of the dyes into photophysical triplet states or by direct contacts between donor and acceptor that lead to quenched dye complexes (Fig. 4 *C*). However, we also identify a slow timescale in the order of 500–2000 μs, which apparently represents slower motional modes of the structure of the DNA. In fact, previous results demonstrated that dsDNA breathing motions exhibit nonexponential dynamics (31) such that our three-state model only provides a simplified description of the true dynamics.

As a second example, we determined the folding-unfolding dynamics of the B-domain of protein A (BDPA) from *Staphylococcus aureus*, a protein that had previously been used to benchmark RASP (22). The particular variant used here (F13W/Y14C/G29A/P57C) has a folding relaxation time of 0.93 ms^{-1} at 2.5 M of the denaturant guanidinium chloride (GdmCl) at 37°C. The protein was labeled at positions 14 and 57 using AlexaFluor488 and AlexaFluor594, and other details of the experiments (buffer, laser intensity, etc.) can be found in Hoffmann et al. (22). Since the experiment was not performed with PIE, we selected the bursts for trBVA based on their FRET value to exclude molecules with inactive acceptor (Fig. 5 *A*, inset). The trBVA trace cannot be described with a single-exponential FRET correlation function (Fig. 5 *A*), and a double-exponential function was required. Whereas the fast rate ($\lambda_1 = 411$ ms^{-1} or 2.4 μs) is associated with the smaller amplitude (36%) and is well in the regime of dye triplet blinking, the slower rate ($\lambda_2 = 0.9$ ms^{-1} or 1.1 ms) dominates the amplitude and indeed corresponds to the timescale observed with RASP (1.4 ms^{-1}) and temperature jump experiments (0.93 ms^{-1}).

In summary, the relaxation times of DNA breathing and of the folding and unfolding of BDPA obtained with trBVA agree well with previous measurements. Compared to our simulations, a very fast relaxation component at timescales of a few microseconds is found in both data sets and might reflect the triplet blinking of our dyes.

CONCLUSION

We presented a time-resolved version of BVA termed trBVA and developed a theoretical framework to apply trBVA in a quantitative manner to smFRET experiments of diffusing molecules. TrBVA is capable of identifying dynamics in biomolecules at timescales from 5 μs up to 5 ms with remarkable accuracy. Using simulated data, we also showed that trBVA can be used in a FRET-resolved manner to identify the FRET values of states that are in exchange. In more complicated cases in which more than two states exchange, FRET-resolved trBVA merely provides qualitative information about the FRET efficiency values of the states. In general, FRET-resolved trBVA is a qualitative tool to understand the complexity of the dynamics at hand.

Finally, we demonstrated the ability of trBVA to identify dynamics in real experiments using the examples of the breathing motions in dsDNA and of fast folding/unfolding kinetics of a protein. We are therefore convinced that trBVA is an excellent addition to the existing toolset of smFRET.

SUPPORTING MATERIAL

Supporting material can be found online at https://doi.org/10.1016/j.bpr.2023.100116.

APPENDIX I

To arrive at Eq. 4, we start from three common assumptions:

1) The total photon rate does not fluctuate in time.
2) The probability, $\epsilon(x(t))$, of observing an acceptor photon is determined by the spatial distance $x(t)$ between donor and acceptor dyes.

3) Dye excitation-emission cycles are fast compared to the inter-photon time.

For an m-photon segment with a given set of photon arrival times $\{t_i\}_{i=1...m}$ resulting from a single trajectory $x(t)$, the probabilities for the individual photons to be detected in the acceptor channel are given by $\{\epsilon_i\}_{i=1...m}$, where $\epsilon_i = \epsilon(x(t_i))$. Our goal is to first compute the first and second moment of the distribution of ϵ for a single trajectory and then to average them over all trajectories. The probability to observe a acceptor photons is therefore given by the Poisson binomial distribution (34) $P_{pb}(a|\{\epsilon_i\}_{i=1}^m)$, which generalizes the ordinary binomial distribution in that the probabilities for individual trials do not need to be equal. The mean of the distribution is known to be $\langle a\rangle = \sum_{i=1}^m \epsilon_i$, and the variance is $\sigma_a^2 = \sum_{i=1}^m \epsilon_i(1-\epsilon_i)$. For the given set of arrival times $\{t_i\}$, we then get the mean and variance of ϵ as

$$\langle\epsilon\rangle_{\{t_i\}} = \langle\frac{a}{m}\rangle_{\{t_i\}} = \frac{1}{m}\sum_{i=1}^m \epsilon_i \tag{I.1}$$

and

$$\sigma_{\{t_i\}}^2 = \langle\epsilon^2\rangle_{\{t_i\}} - \langle\epsilon\rangle_{\{t_i\}}^2 = \frac{1}{m^2}\sum_{i=1}^m \epsilon_i(1-\epsilon_i). \tag{I.2}$$

For the second moment, we can then write the following:

$$\langle\epsilon^2\rangle_{\{t_i\}} = \frac{1}{m^2}\left[\sum_{i=1}^m \epsilon_i(1-\epsilon_i) + \left(\sum_{i=1}^m \epsilon_i\right)^2\right]$$

$$= \frac{1}{m^2}\left[\sum_{i=1}^m \epsilon_i + \sum_{i\neq j}\epsilon_i\epsilon_j\right]. \tag{I.3}$$

We now need to average over all sets of arrival times. To this end, consider photon segments that have a fixed duration t between the first and last photon, whereas all $m-2$ photons in between the first and last photon have random arrival times. For the moment, we only consider a specific trajectory $x(t')$ of a molecule, but we will average over all trajectories at a later stage. To compute the total probability of obtaining a acceptor photons in a photon segment, we need to average over all possible arrival times $\{t_i\}_{i=1...m}$. Note that the arrival times are not an ordered set. Whereas the arrival times are fixed for the first and last photon ($t_1 = 0$ and $t_m = t$), the arrival times of the remaining $m-2$ photons are independently and uniformly distributed random values between 0 and t. The probability density function of such sets $\{t_i\}_{i=1...m}$ is given by

$$P\left(\{t_i\}_{i=1}^m|t\right) = \frac{4}{t^{m-2}}\delta(t_1-0)\delta(t_m-t) \text{ with}$$

$$\int_0^t\cdots\int_0^t P\left(\{t_i\}_{i=1}^m|t\right)\, dt_1...dt_m = 1. \tag{I.4}$$

As mentioned above, the number of acceptor photons for a given set $\{t_i\}$ obeys Poisson binomial statistics. The mean FRET efficiency for the trajectory $x(t')$ is then given by

$$\langle\epsilon\rangle(m,t,x(t')) = \int_0^t\cdots\int_0^t \langle\epsilon\rangle_{\{t_i\}}\, P\left(\{t_i\}_{i=1}^m|t\right)\, dt_1...dt_m$$

$$= \int_0^t\cdots\int_0^t\left(\frac{1}{m}\sum_{i=1}^m \epsilon(x(t_i))\right)$$

$$P\left(\{t_i\}_{i=1}^m|t\right)\, dt_1...dt_m = \frac{1}{m}\sum_{i=1}^m\int_0^t\cdots$$

$$\int_0^t \epsilon(x(t_i))\, P\left(\{t_i\}_{i=1}^m|t\right)\, dt_1...dt_m$$

$$= \frac{1}{m}\left[\epsilon(x(0)) + \epsilon(x(t)) + \frac{1}{t^{m-2}}\sum_{i=2}^{m-1}\right.$$

$$\left.\int_0^t\cdots\int_0^t \epsilon(x(t_i))\ dt_2...dt_{m-1}\right] = \frac{1}{m}$$

$$\left[\epsilon(x(0)) + \epsilon(x(t)) + (m-2)\frac{1}{t}\int_0^t dt'\epsilon(x(t'))\right] \tag{I.5}$$

Similarly, with the use of Eq. I.3, the second moment is

$$\langle\epsilon^2\rangle(m,t,x(t')) = \int_0^t\cdots\int_0^t\langle\epsilon^2\rangle_{\{t_i\}}\, P\left(\{t_i\}_{i=1}^m|t\right)\, dt_1...$$

$$dt_m = \frac{1}{m^2}\int_0^t\cdots\int_0^t\left[\sum_{i=1}^m\epsilon(x(t_i)) + \sum_{i\neq j}\epsilon(x(t_i))\epsilon\left(x(t_j)\right)\right]$$

$$P\left(\{t_i\}_{i=1}^m|t\right)\, dt_1...dt_m = \frac{1}{m}\langle\epsilon\rangle(m,t,x(t')) + \frac{1}{m^2}\frac{1}{t^{m-2}}$$

$$\sum_{i\neq j}^m\int_0^t\cdots\int_0^t \epsilon(x(t_i))\epsilon\left(x(t_j)\right)4\delta(t_1-0)\delta(t_m-t)$$

$$dt_1...dt_m = \frac{1}{m}\langle\epsilon\rangle(m,t,x(t')) + \frac{1}{m^2}\left\{2[\epsilon(x(0))\epsilon(x(t))]\right.$$

$$+2(m-2)\frac{1}{t}\int_0^t dt'[\epsilon(x(0))\epsilon(x(t'))] + 2(m-2)\frac{1}{t}$$

$$\int_0^t dt'[\epsilon(x(t))\epsilon(x(t'))] + (m-2)(m-3)\frac{1}{t^2}\int_0^t\int_0^t$$

$$\left.dt'dt''[\epsilon(x(t'))\epsilon(x(t''))]\right\} \tag{I.6}$$

At this stage, we average both results I.5 and I.6 over the ensemble of distance trajectories and denote this average as $\langle \ldots \rangle_x$. In this notation, the FRET efficiency averaged over all trajectories $\langle \epsilon(x(t)) \rangle_x$ and $\delta\epsilon(x(t))$ are then written as

$$\langle \epsilon \rangle_x \equiv \langle \epsilon(x(t)) \rangle_x ;\ \delta\epsilon(x(t)) = \epsilon(x(t)) - \langle \epsilon \rangle_x \text{ with } \langle \delta\epsilon(x(t)) \rangle_x = 0 \tag{I.7}$$

After averaging, the result for the mean FRET efficiency is

$$\langle \epsilon \rangle_x(m,t) = \langle \langle \epsilon \rangle(m,t,x(t')) \rangle_x = \frac{1}{m}\left[\langle \epsilon(x(0)) \rangle_x + \langle \epsilon(x(t)) \rangle_x + (m-2)\frac{1}{t}\int_0^t dt' \langle \epsilon(x(t')) \rangle_x \right] = \langle \epsilon \rangle_x \tag{I.8}$$

Similarly, averaging Eq. I.6 gives

$$\langle \epsilon^2 \rangle_x(m,t) = \langle \langle \epsilon^2 \rangle(m,t,x(t')) \rangle_x = \frac{1}{m}\langle \epsilon \rangle_x + \left(1 - \frac{1}{m}\right)\langle \epsilon \rangle_x^2 + \frac{1}{m^2}\Bigg[2\langle \delta\epsilon(x(0))\delta\epsilon(x(t')) \rangle_x + 4(m-2)\frac{1}{t}\int_0^t dt' \langle \delta\epsilon(x(0))\delta\epsilon(x(t')) \rangle_x + (m-2)(m-3)\frac{2}{t^2}\int_0^t dt'(t-t')\langle \delta\epsilon(x(0))\delta\epsilon(x(t')) \rangle_x \Bigg] \tag{I.9}$$

The variance of segments with length m and time duration t is then

$$\sigma_x^2(m,t) = \langle \epsilon^2 \rangle_x(m,t) - \langle \epsilon \rangle_x^2 = \frac{\langle \epsilon \rangle_x (1 - \langle \epsilon \rangle_x)}{m} + \Delta s^2(m,t), \tag{I.10}$$

where the first term is the variance of a binomial distribution. The second term is the t-specific excess variance that contains information about the conformational fluctuations and with $g(t) = \langle \delta\epsilon(0)\delta\epsilon(t) \rangle_x$ is given by

$$\Delta s^2(m,t) = \frac{1}{m^2}\left[2\,g(t) + \frac{4(m-2)}{t}\int_0^t g(t')dt' + \frac{2(m-2)(m-3)}{t^2}\int_0^t (t-t')g(t')dt' \right], \tag{I.11}$$

which is identical to Eq. 4 in the main text. It is noteworthy that the above result is similar to the result obtained by Gopich and Szabo for the case of fixed time bins instead of variable segment lengths (16). The main difference is that all photons in a fixed time bin are randomly distributed, whereas in BVA, the arrival times of the first and the last photon in a segment are fixed. When relaxing this constraint, the probability density function in Eq. I.4 becomes

$$P\left(\{t_i\}_{i=1}^m \middle| t\right) = \frac{1}{t^m}, \tag{I.12}$$

and solving the integrals in I.5 and I.6, the t-specific excess variance becomes

$$\Delta s^2(m,t) = \left(1 - \frac{1}{m}\right)\frac{2}{t^2}\int_0^t (t-t')g(t')dt', \tag{I.13}$$

which is similar to Eq. 3.8 in Gopich and Szabo (16) if only bins of time t with m photons are considered.

APPENDIX II

Here we provide the formulas for the t-specific excess variance $\Delta s^2(m,t)$ used for data fitting with the two-state and three-state model. As outlined in Eq. 7, the correlation function of a two-state model is given by $g_{2-State}(t) = a\,e^{-\lambda t}$, where $a = \frac{k_{12}k_{21}}{k_{12}+k_{21}}(\epsilon_1 - \epsilon_2)^2$ and $\lambda = k_{12} + k_{21}$. Similarly, the correlation function for a three-state model can be obtained from Eq. 6 using the appropriate rate matrix $\mathbf{K}$ and the diagonal matrix containing the FRET efficiencies of the three states $\boldsymbol{\epsilon}$. For instance, for the model shown in Fig. 3 C (top), we have

$$\mathbf{K} = \begin{pmatrix} -k_{12} & k_{21} & 0 \\ k_{12} & -(k_{21}+k_{23}) & k_{32} \\ 0 & k_{23} & -k_{32} \end{pmatrix} \text{ and } \boldsymbol{\epsilon} = \begin{pmatrix} \epsilon_1 & 0 & 0 \\ 0 & \epsilon_2 & 0 \\ 0 & 0 & \epsilon_3 \end{pmatrix} \tag{II.1}$$

with the equilibrium state vector

$$\mathbf{p_{eq}} = (k_{21}k_{32} + k_{12}k_{32} + k_{12}k_{23})^{-1} \begin{pmatrix} k_{21}k_{32} & k_{12}k_{32} & k_{12}k_{23} \end{pmatrix}. \tag{II.2}$$

The rate matrix $\mathbf{K}$ has two nonzero eigenvalues λ_1 and λ_2, and the correlation function can be written as $g_{3-State}(t) = a_1 e^{-\lambda_1 t} + a_2 e^{-\lambda_2 t}$. Inserting these expressions in Eq. 4 and solving the integrals gives

$$\Delta s^2_{2-State}(m,t) = \frac{1}{m^2}\left\{2ae^{-\lambda t} + 4(m-2)\frac{a(1-e^{-\lambda t})}{\lambda t} + 2(m-2)(m-3)\frac{a(e^{-\lambda t}-1+\lambda t)}{\lambda^2 t^2}\right\} \tag{II.3}$$

$$\Delta s^2_{3-State}(m,t) = \frac{1}{m^2}\left\{2\left[a_1 e^{-\lambda_1 t} + a_2 e^{-\lambda_2 t}\right] + 4(m-2)\left[\frac{a_1(1-e^{-\lambda_1 t})}{\lambda_1 t} + \frac{a_2(1-e^{-\lambda_2 t})}{\lambda_2 t}\right] + 2(m-2)(m-3)\left[\frac{a_1(e^{-\lambda_1 t}-1+\lambda_1 t)}{\lambda_1^2 t^2} + \frac{a_1(e^{-\lambda_2 t}-1+\lambda_2 t)}{\lambda_2^2 t^2}\right]\right\}. \tag{II.4}$$

APPENDIX III

For dynamics that are slow compared to the duration of a segment t, we can approximate the correlation function by a constant number $\langle\delta\epsilon(0)\delta\epsilon(t')\rangle_x \approx \langle\delta\epsilon^2\rangle_x$ for $t' \in (0,t)$. In this case, the t-specific excess variance (I.11) simplifies to

$$\Delta s^2(m,t) = \frac{m-1}{m}\langle\delta\epsilon^2\rangle_x = \left(1-\frac{1}{m}\right)\sigma^2_\epsilon, \tag{III.1}$$

where $\sigma^2_\epsilon \equiv \langle\delta\epsilon^2\rangle_x = \langle\epsilon^2\rangle_x - \langle\epsilon\rangle^2_x$ is the variance of the FRET efficiency over all conformational states. Note that Eq. III.1 is an increasing function with m. Following Eq. I.10, the total t-specific variance in the static case is then

$$\begin{aligned}\sigma^2_x(m,t) &= \frac{\langle\epsilon\rangle_x(1-\langle\epsilon\rangle_x)}{m} + \left(1-\frac{1}{m}\right)\sigma^2_\epsilon \\ &= \sigma^2_\epsilon + \frac{1}{m}\left(\langle\epsilon\rangle_x - \langle\epsilon\rangle^2_x - \sigma^2_\epsilon\right) \\ &= \sigma^2_\epsilon + \frac{\langle\epsilon(1-\epsilon)\rangle_x}{m}.\end{aligned} \tag{III.2}$$

From the last expression, it is clear that the total t-specific variance is a decaying function of m (third term in III.2), as expected based on the central limit theorem. Using the expression $\langle\epsilon\rangle_x(1-\langle\epsilon\rangle_x)/m$ to estimate shot noise using binomial statistics, which is only correct in the absence of heterogeneity, causes the factor $(1-m^{-1})$ in the t-specific excess variance (III.1). A better estimate for shot noise in the static case is therefore $\langle\epsilon(1-\epsilon)\rangle_x/m$.

The other limit is given when the timescale of conformational dynamics (τ) is much faster than the interphoton time. Hence, the time duration of any segment (t) is much longer than τ or, equivalently, $P(t|m) \ll 1$ for $t \sim \tau$. When evaluating the integral in Eq. 5, the distribution $P(t|m)$ has nonzero weights only for those values of $\Delta s^2(m,t)$ for which $g(t) \sim 0$, and therefore $\Delta s^2(m,t) \sim 0$. Correspondingly, Eq. 5 evaluates to $S^2 \sim 0$, which is the lower boundary of S^2.

APPENDIX IV

To judge the accuracy of the parameters that can be obtained from trBVA traces, we provide a lower limit for the amplitude of the autocorrelation function. In a two-state system, the amplitude of the FRET autocorrelation function will increase with $\Delta\epsilon^2 = (\epsilon_2 - \epsilon_1)^2$ (see Eq. 7, Fig. S1 *A*). Clearly, the higher the FRET-separation of the two states is, the higher is the amplitude of the autocorrelation function. A lower limit of $\Delta\epsilon$ should be given by shot noise. For simplicity, we assume that the two states exchange with slow dynamics compared to the diffusion time through the confocal volume. In addition, we assume that their shot noise variance is identical and given by σ. To distinguish the two states in a FRET efficiency histogram, the separation between the states should exceed the combined shot noise variance of both states. We therefore require

$$\Delta\epsilon > 2\sigma. \tag{IV.1}$$

From this expression, we can get a lower limit for the amplitude of the FRET autocorrelation function. The amplitude is given $a = k_{12}k_{21}\Delta\epsilon^2/\lambda^2$, where $\lambda = k_{12} + k_{21}$. Given a value for the difference $\Delta\epsilon$, the best possible case (the highest amplitude) would be at $k_{12} = k_{21}$. Any other combination of k_{12} and k_{21} would result in even smaller amplitudes. With $k_{12} = k_{21}$, the amplitude reduces to $a = \Delta\epsilon^2/4$, and with Eq. IV.1, we would require

$$a > \sigma^2. \tag{IV.2}$$

Importantly, even if σ of a single state cannot be reliably determined because of a high overlap between the states or because of fast exchange dynamics, the above inequality would still provide a lower limit for the amplitude below which a determination of the FRET autocorrelation function becomes unreliable. For instance, for a measured FRET distribution centered at $\epsilon = 0.5$ and a threshold of 100 photons, one would ideally like to have an amplitude of $a > 0.5^2/100 = 2.5\,10^{-3}$ and $\Delta\epsilon > 2\sqrt{0.5^2/100} = 0.1$. We tested this estimate with simulations assuming identical kinetic rates ($k_{12} = k_{21} = 1\ ms^{-1}$) but different values of $\Delta\epsilon$ (Fig. S1). The apparent kinetic rate λ is obtained with good accuracy down to a value of $\Delta\epsilon = 0.2$. However at $\Delta\epsilon \approx 0.1$ and at an amplitude of $a \approx 3.5\,10^{-3}$, i.e., close to our accuracy estimates of $\Delta\epsilon > 0.1$ and $a > 2.5\,10^{-3}$, the fitted rate exceeds the ground truth threefold

(Fig. S1 *B*), indicating that the parameters of the FRET autocorrelation function cannot be reliably determined. In addition, we tested the sensitivity of trBVA to the photon threshold used to identify bursts. Simulations show that the photon threshold has no impact on the determined kinetic rates from trBVA as long as the FRET efficiency separation between the states fulfills Eq. IV.1 (Fig. S1 *B*, inset). Yet, at an extremely low separation $\Delta\epsilon \approx 0.1$, a doubling of the photon threshold from $T = 100$ to $T = 200$ indeed lowers the discrepancy between fitted rate and ground truth.

AUTHOR CONTRIBUTIONS

I.T. and H.H. performed research. D.N. and D.E.M. provided tools and input to the theory. H.H. designed research. H.H. wrote the manuscript with contributions from all authors.

ACKNOWLEDGMENTS

We particularly thank Benjamin Schuler for helpful comments on the manuscript and the smFRET data of BDPA. This work was supported by a grant of the European Research Council (grant no. 864578) and by a research grant from the Corrine Koshland Instrument Fund (for incumbent of CDC 10673) to H.H. D.E.M. was supported by the Robert A. Welch Foundation (grant no. F-1514) and by the National Science Foundation (grant no. CHE 1955552).

DECLARATION OF INTERESTS

The authors declare no competing interest.

REFERENCES

1. Schuler, B., and H. Hofmann. 2013. Single-molecule spectroscopy of protein folding dynamics-expanding scope and timescales. *Curr. Opin. Struct. Biol.* 23:36–47.
2. Schuler, B., A. Soranno, ..., D. Nettels. 2016. Single-molecule FRET spectroscopy and the polymer physics of unfolded and intrinsically disordered proteins. *Annu. Rev. Biophys.* 45:207–231.
3. Barth, A., O. Opanasyuk, ..., C. A. M. Seidel. 2022. Unraveling multi-state molecular dynamics in single-molecule FRET experiments. I. Theory of FRET-lines. *J. Chem. Phys.* 156, 141501.
4. Opanasyuk, O., A. Barth, ..., C. A. M. Seidel. 2022. Unraveling multi-state molecular dynamics in single-molecule FRET experiments. II. Quantitative analysis of multi-state kinetic networks. *J. Chem. Phys.* 157, 031501.
5. Hellenkamp, B., S. Schmid, ..., T. Hugel. 2018. Precision and accuracy of single-molecule FRET measurements-a multi-laboratory benchmark study. *Nat. Methods.* 15:669–676.
6. Hofmann, H., and W. Zheng. 2022. Single-molecule fluorescence spectroscopy of intrinsically disordered proteins. *In* Fluorescence Spectroscopy and Microscopy in Biology. R. Šachl and M. Amaro, eds Springer International Publishing.
7. Kalinin, S., A. Valeri, ..., C. A. M. Seidel. 2010. Detection of structural dynamics by FRET: A photon distribution and fluorescence lifetime analysis of systems with multiple states. *J. Phys. Chem. B.* 114:7983–7995.
8. Gopich, I. V., and A. Szabo. 2008. Theory of Photon Counting in Single Molecule Spectroscopy. World Scientific Publishing Co. Pte. Ltd..
9. Chung, H. S., and W. A. Eaton. 2013. Single-molecule fluorescence probes dynamics of barrier crossing. *Nature.* 502:685–688.
10. Chung, H. S., S. Piana-Agostinetti, ..., W. A. Eaton. 2015. Structural origin of slow diffusion in protein folding. *Science.* 349:1504–1510.
11. Gopich, I. V., and A. Szabo. 2009. Decoding the pattern of photon colors in single-molecule FRET. *J. Phys. Chem. B.* 113:10965–10973.
12. Gopich, I. V. 2015. Accuracy of maximum likelihood estimates of a two-state model in single-molecule FRET. *J. Chem. Phys.* 142, 034110.
13. Pirchi, M., R. Tsukanov, ..., E. Nir. 2016. Photon-by-photon hidden Markov model analysis for microsecond single-molecule FRET kinetics. *J. Phys. Chem. B.* 120:13065–13075.
14. Aviram, H. Y., M. Pirchi, ..., G. Haran. 2018. Direct observation of ultrafast large-scale dynamics of an enzyme under turnover conditions. *Proc. Natl. Acad. Sci. USA.* 115:3243–3248.
15. Harris, P. D., A. Narducci, ..., E. Lerner. 2022. Multi-parameter photon-by-photon hidden Markov modeling. *Nat. Commun.* 13:1000–1012.
16. Gopich, I., and A. Szabo. 2005. Theory of photon statistics in single-molecule Förster resonance energy transfer. *J. Chem. Phys.* 122, 14707.
17. Ghosh, A., N. Karedla, ..., J. Enderlein. 2018. Fluorescence lifetime correlation spectroscopy: Basics and applications. *Methods.* 140–141:32–39.
18. Felekyan, S., S. Kalinin, ..., C. A. M. Seidel. 2012. Filtered FCS: species auto- and cross-correlation functions highlight binding and dynamics in biomolecules. *ChemPhysChem.* 13:1036–1053.
19. Ishii, K., and T. Tahara. 2012. Extracting decay curves of the correlated fluorescence photons measured in fluorescence correlation spectroscopy. *Chem. Phys. Lett.* 519–520:130–133.
20. Ishii, K., and T. Tahara. 2013. Two-dimensional fluorescence lifetime correlation spectroscopy. 1. Principle. *J. Phys. Chem. B.* 117:11414–11422.
21. Ishii, K., and T. Tahara. 2013. Two-dimensional fluorescence lifetime correlation spectroscopy. 2. Application. *J. Phys. Chem. B.* 117:11423–11432.
22. Hoffmann, A., D. Nettels, ..., B. Schuler. 2011. Quantifying heterogeneity and conformational dynamics from single molecule FRET of diffusing molecules: recurrence analysis of single particles (RASP). *Phys. Chem. Chem. Phys.* 13:1857–1871.
23. Wiggers, F., S. Wohl, ..., H. Hofmann. 2021. Diffusion of a disordered protein on its folded ligand. *Proc. Natl. Acad. Sci. USA.* 118, e2106690118.
24. Saurabh, A., M. Fazel, ..., S. Pressé. 2023. Single-photon smFRET. I: Theory and conceptual basis. *Biophys. Rep.* 3, 100089.
25. Saurabh, A., M. Safar, ..., S. Pressé. 2023. Single-photon smFRET: II. Application to continuous illumination. *Biophys. Rep.* 3, 100087.
26. Safar, M., A. Saurabh, ..., S. Pressé. 2022. Single-photon smFRET. III. Application to pulsed illumination. *Biophys. Rep.* 2, 100088.
27. Torella, J. P., S. J. Holden, ..., A. N. Kapanidis. 2011. Identifying molecular dynamics in single-molecule FRET experiments with burst variance analysis. *Biophys. J.* 100:1568–1577.
28. Crank, J. 1975. The Mathematics of Diffusion, Second edition. Oxford University Press.
29. Müller, B. K., E. Zaychikov, ..., D. C. Lamb. 2005. Pulsed interleaved excitation. *Biophys. J.* 89:3508–3522.

30. Schuler, B. 2007. Application of single molecule Förster resonance energy transfer to protein folding. *Methods Mol. Biol.* 350:115–138.

31. Altan-Bonnet, G., A. Libchaber, and O. Krichevsky. 2003. Bubble dynamics in double-stranded DNA. *Phys. Rev. Lett.* 90:138101–138104.

32. Lando, D., S. G. Haroutiunian, …, A. A. Akhrem. 1994. Theoretical and experimental study of DNA helix-coil transition in acidic and alkaline medium. *J. Biomol. Struct. Dyn.* 12:355–366.

33. Rosenblum, G., N. Elad, …, H. Hofmann. 2021. Allostery through DNA drives phenotype switching. *Nat Comms.* 12:2967.

34. Nedelman, J., and T. Wallenius. 1986. Bernoulli trials, Poisson trials, surprising variances, and Jensen's inequality. *Am. Stat.* 40:286–289.

Biophysical Reports, Volume *3*

Supplemental information

Time-resolved burst variance analysis

Ivan Terterov, Daniel Nettels, Dmitrii E. Makarov, and Hagen Hofmann

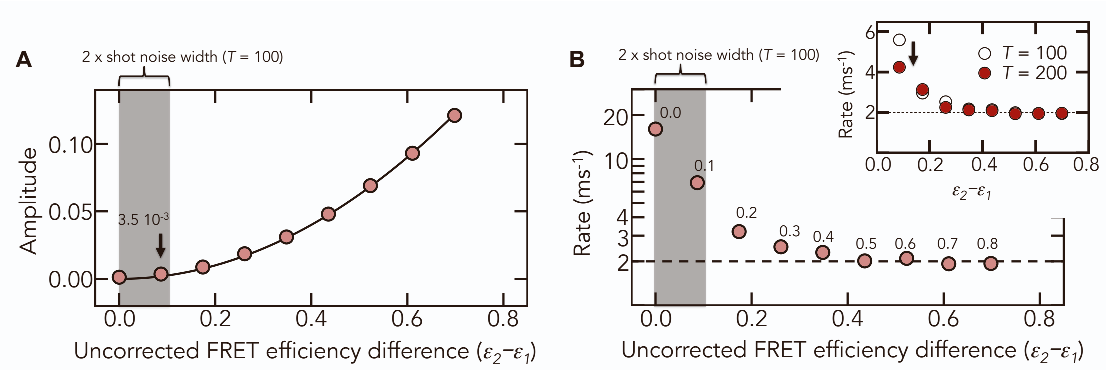

Figure S1. Simulations of 2-states in exchange with different FRET efficiency separation. (A) Amplitudes of the FRET autocorrelation function as function of the uncorrected FRET efficiency separation between the two states. The exchange rates were $k_{12} = k_{21} = 1ms^{-1}$ and the positions of the states were at the corrected FRET efficiencies $E_1 = 1/2 - \Delta E$ and $E_2 = 1/2 + \Delta E$. The solid line is the expected increase based on eq. 7 in the main text. The gray area indicates the regime of strong overlap between the two FRET states (see eq. VI.1). **(B)** Kinetic rates as function of the uncorrected FRET efficiency separation between the states obtained from the simulations shown in A. The dashed line indicates the ground truth ($k_{12} + k_{21} = 2ms^{-1}$). Numbers above the symbols indicate the corrected FRET efficiency separation ($2\Delta E$). The gray area indicates the regime of strong overlap between the two FRET states (see eq. VI.1). Inset: Dependence of the kinetic rate on the choice of the burst identification threshold T (indicated).
